# An integrated method for cell isolation and migration on a chip

**DOI:** 10.1038/s41598-017-08661-z

**Published:** 2017-08-21

**Authors:** Xiaoqing Lv, Zhaoxin Geng, Zhiyuan Fan, Shicai Wang, WeiHua Pei, Hongda Chen

**Affiliations:** 1 State Key Laboratory of Integrated Optoelectronics, Institute of Semiconductors, Chinese Academy of Sciences, Beijing, China; 2 School of Information Engineering, Minzu University of China, Beijing, China; 3 State Key Laboratory of crystal materials, Shandong University, Jinan, China; 4 University of Chinese Academy of Sciences, Beijing, China

## Abstract

Tumour cell migration has an important impact on tumour metastasis. Magnetic manipulation is an ascendant method for guiding and patterning cells. Here, a unique miniaturized microfluidic chip integrating cell isolation and migration assay was designed to isolate and investigate cell migration. The chip was fabricated and composed of a magnet adapter, a polytetrafluoroethylene(PDMS) microfluidic chip and six magnetic rings. This device was used to isolate MCF-7 cells from MDA-MB-231-RFP cells and evaluate the effects of TGF-β on MCF-7 cells. First, the two cell types were mixed and incubated with magnetic beads modified with an anti-EpCAM antibody. Then, they were slowly introduced into the chip. MCF-7 cells bond to the magnetic beads in a ring-shaped pattern, while MDA-MB-231-RFP cells were washed away by PBS. Cell viability was examined during culturing in the micro-channel. The effects of TGF-β on MCF-7 cells were evaluated by migration distance and protein expression. The integrated method presented here is novel, low-cost and easy for performing cell isolation and migration assay. The method could be beneficial for developing microfluidic device applications for cancer metastasis research and could provide a new method for biological experimentation.

## Introduction

Cell migration is a key process in embryonic morphogenesis, tissue repair and regeneration, cancer metastasis, mental retardation, atherosclerosis, and arthritis^1^. In particular, metastasis, which involves primary tumor migrating to secondary metastatic sites, is a major reason for breast cancer-associated mortality. Therefore, investigation of cancer cell migration has recently received intensive attention^2^. Nevertheless, the mechanisms of cancer cell migration are still unknown because the invasion behaviours of primary breast cancer cells and their potential to metastasize are complex processes^3^. There are many cell subtypes in tumours, and only few of them promote tumour metastasis^4^. Isolating cells from complex, heterogeneous mixtures is a critical task in many areas of biology, biotechnology and medicine^5^. It is necessary for enriching or purifying cell samples into well-defined populations in many diagnostic and therapeutic practices^6^. The first cell sorting method was fluorescence-activated cell sorting (FACS), which is capable of multiplexd detections and analyses. There are many novel methods for isolating cells using microfluidic chip including magnetic-based cell sorting^7^, surface acoustic waves^8^, dielectrophoresis^9^, hydrodynamic force^10^, and filtration^11^. Among these novel methods, magnetic-based cell sorting is widely used due to its rapid and label-free sorting process.

Recent studies have shown that the epithelial–mesenchymal transition (EMT) is important for the invasion and metastasis of breast cancer cells^12^. Understanding the mechanisms underlying cell migration is very important for cellular transplantation and the development of new therapeutic strategies for controlling invasive tumour cells^13^. The gold standard for cell migration assays include Boyden chamber and wound-healing assays, both of which are easily performed with short experimental times and a stable extracellular environment, but they have some inherent limitations^14^. To date, with the development of microfabrication technology, microfluidic chips have emerged as a revolutionary, novel platform for many applications in cell biology, bionic organs, cell and particle sorting and disease detection^15–21^. Microfluidics-based wound-healing assays have been proposed as effective tools for studying cell migration, which can mimic *in vivo* microenvironments more closely than conventional wound-healing assays^22–25^. Advances in microfluidics techniques have allowed for cell migration assays on a simple microfluidic chip by replicating the traditional biological laboratory^26–30^. For example, sheer flow in a microchannel is one of the most widely used methods for developing a scratch^11^; it is a beneficial and mimicked method using advantages of microfluidic chip, though sheer flow is hard to control during the experiment. Moreover, cell exposure to sheer stress affects protein expression and cytoskeleton. Magnetic-based devices are another approach to manipulate cell migration^31^. Magnetic-based techniques were used because they are convenient to control during the experiment with simple components. By binding with nanoparticles, the cells can respond to changes in the magnetic field and can be manipulated with a magnet. A novel cell migration approach that uses a magnet to control cell wounding has been established, and now, a commercial product related to this method has been sold by Nano3D Biosciences, Inc^32^. However, the price of a complete set of experimental materials is expensive. Another commercial cell migration device is the Radius™ 96-Well Cell Migration Assay, which is high-throughput. It provides a robust *in vitro* system to measure 2D cell migration, screen potential inhibitors and study cytoskeleton reorganization events. However, in daily experiments, this high-throughput device is not commonly used because three to six sets of experiments were enough to adapt most of the experimental requests. Furthermore, there is no device that can integrate cell isolation and a migration assay on a chip. Hence, a low-cost and damage-free method to achieve cell isolation and a migration assay on a chip is of great potential for experimentation. The method presented here can capture cell subtypes with specific protein expression with antibody-modified magnetic beads and then investigate the migration behaviours of this subtype of cells.

This study describes an integrated chip that combines cell isolation and a migration assay. In this assay, we use a magnetic-based method to isolate two cell subtypes which express different membrane proteins on a chip, and performed a cell migration assay on the chip. A mixture of MCF-7 and MDA-MB-231-RFP cells was incubated with magnetic beads modified with an anti-EpCAM antibody. The cells adopted a ring-shaped pattern due to the magnetic force after being loaded into the channel. Following a wash step, most of the MDA-MB-231-RFP cells, which express low EpCAM protein level, were washed away. When the magnet was removed, MCF-7 cells migrated to the ring’s centre due to different stimuli. The specificity of the MCF-7 cells from the mixture of MDA-MB-231-RFP cells demonstrates the basic function of this system. Moreover, TGF-β, which is a main factor that accelerates MCF-7 migration, promotes the critical EMT mesenchymal marker vimentin that directly induces the migration of MCF-7 cells aided by this integrated chip. As a platform technology, the integrated cell isolation and migration chip presented here can be readily applicable to investigate cell subtype migration abilities in a mixture of complicated tumour cells.

## Results and Discussion

### Construction of the device

**Figure 1:**
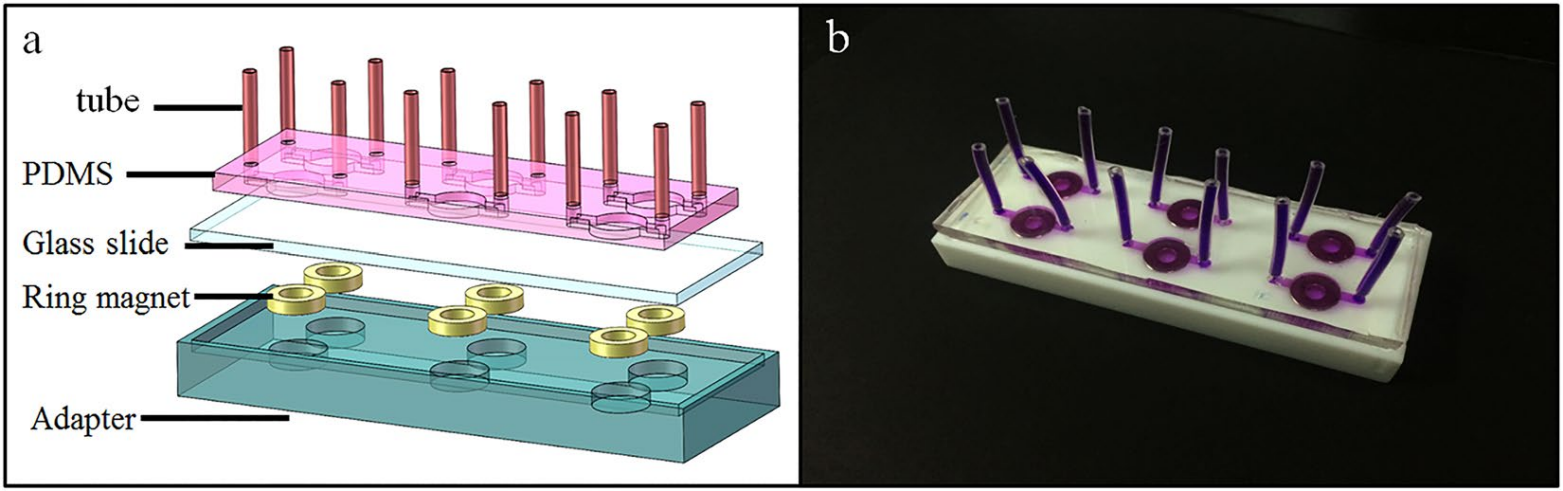
Images of the integrated cell isolation and migration device. (**a**) Schematic diagram of the device that is composed of a PDMS microfluidic chip with six cell culture channels, a glass slide, six ring magnets and an adapter. (**b**) Photograph of the real device used in this experiment. Purple ink is used to display the microfluidic channels.

An integrated method for cell isolation and migration on a microfluidic chip was proposed to isolate specific cells from a cell mixture sample and investigate these cells’ migration behaviour under different stimuli. Construction of the device is shown in Fig. 1. The device was easily fabricated in the laboratory. General use materials were utilized to design the microfluidic chip. For example, the magnet adapter was fabricated using PDMS, which has many advantages, including stability and high temperature sterilization. The microchannel was made from PDMS, which is nontoxic to cells and transparent for microscopy observation during the culturing and staining procedures. The magnet is a commercial product and easily obtained. The channel radius was fitted well within the out-radius of the magnet ring and can promote the cells to form a ring-shaped pattern. A hollow cellular layer was used for the “wound” so that the cells had enough space to migrate when treated with various stimuli.

Moreover, the microfluidic chip can provide various information tests about the cells aside from the migration assay. For example, the cells in the channel can be directly stained to investigate their protein expression for deeper mechanism analyses. Compared with a traditional wound-healing assay, the chip could potentially serve as a low-cost method. Only 50 μL of cell culture medium is needed for one channel, and the 6-well culture cluster can complete a wound-healing assay with 2 mL of medium. At the same time, the amount of antibody, dye, wash buffer and other buffer in the staining procedure are also remarkably reduced.

### Working principle of the chip

**Figure 2:**
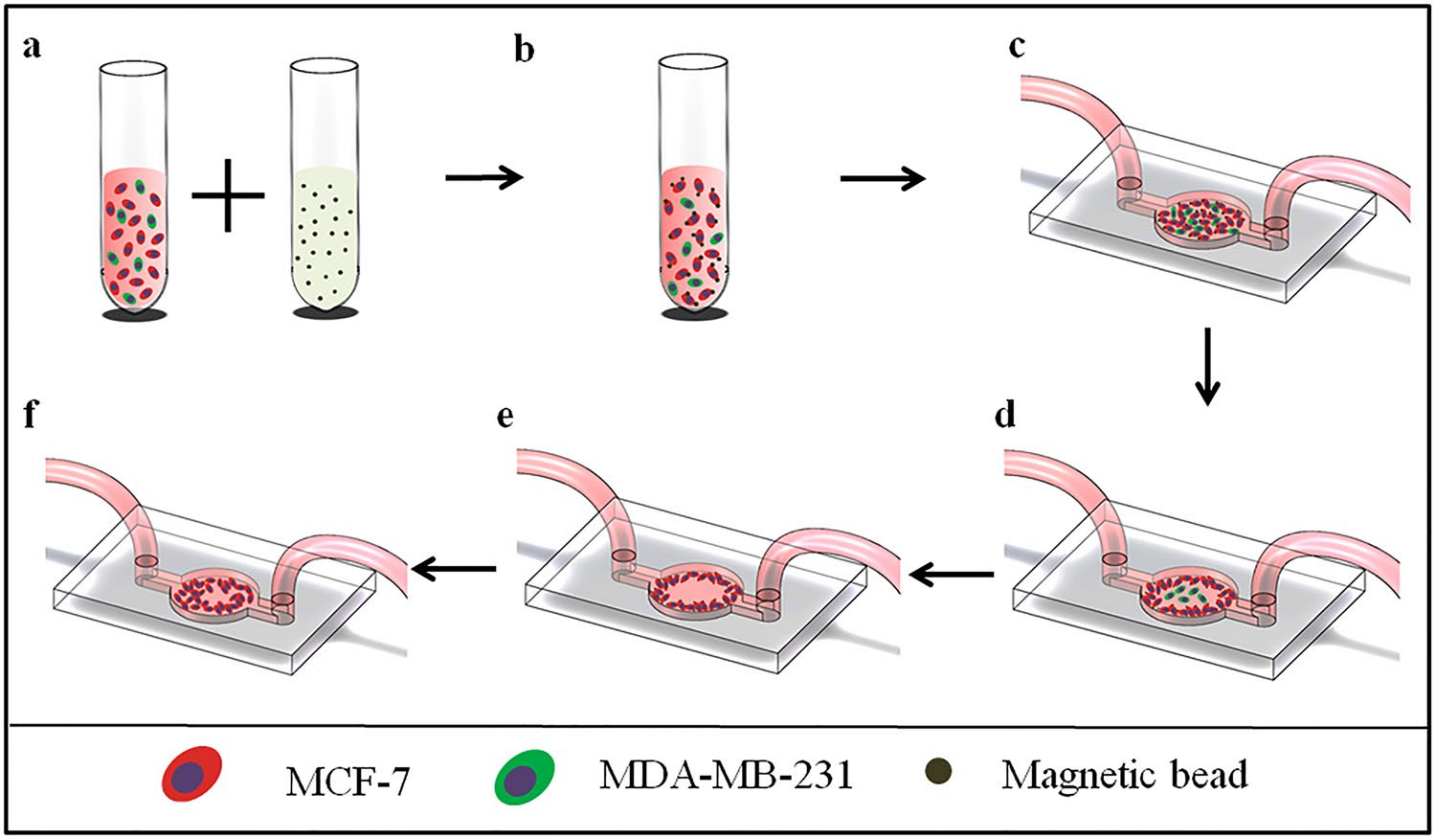
The cell isolation and migration assays. (**a**) MCF-7 and MDA-MB-231 cells were mixed and incubated with anti-EpCAM antibody-modified magnetic beads. (**b**) After incubation for 20 mins, the cell mixture began to bind with the magnetic beads and was then washed with PBS twice. (**c**) The microfluidic chip was pre-treated with fibronectin at a concentration of 50 ng/mL and washed with cell culture medium before the cell mixture was introduced into the channel. The chip was shaken after injecting the cells to ensure the cell suspension was homogeneous. (**d**) MCF-7 cells that bound to the magnetic beads formed a ring-shaped pattern when the chip was placed on the magnet. (**e**) MDA-MB-231-RFP cells were washed away with PBS because they express low EpCAM protein level. This left the MCF-7 cells in a ring-shaped pattern in the channel. (**f**) Then, the cells migrated to the centre of the channel under different stimuli.

Schematics of the experimental process are shown in Fig. 2. The mechanism is similar to that of a traditional wound-healing assay, in which cells migrate close to centre of the scratch. First, MCF-7 and MDA-MB-231-RFP cells were mixed and incubated with anti-EpCAM antibody-modified magnetic beads (Fig. 2a). The MCF-7 cells bound to the magnetic beads and became magnetized via incubation with epithelial enrich magnetic beads (Figs 2b and S2) because MDA-MB-231-RFP cells express low EpCAM protein level. The diameter of the bead is 4 μm. The chip was connected with inlet and outlet tubes. Before use, the chip was pre-treated with fibronectin to improv cell adherence and washed with cell culture medium. Then, the cell mixture was loaded into the channel, followed by shaking for a few minutes to promote the cells to distribute uniformly (Fig. 2c). Next, the chip was placed onto the magnet adapter. The MCF-7 cells moved into a circular ring-shape under the magnet force, forming a scratch (Fig. 2d). After the circular ring formed, the MDA-MB-231-RFP cells were washed away using PBS (Fig. 2e). Then, the chip and adapter were placed in an incubator at 37 °C with 5% CO_2_ to make the magnetic cells adhere to the culture surface. After a 6 h incubation, the adapter was removed away from the chip, and the cells continued to grow normally on the chip. Then, fresh medium with TGF-β at a concentration of 0, 5, or 10 ng/mL was individually added to each channel (Fig. 2f). The medium was replaced each day until the staining experiment was completed to prevent nutrient deprivation and growth factor depletion.

### Cell viability in the micro-channel

**Figure 3:**
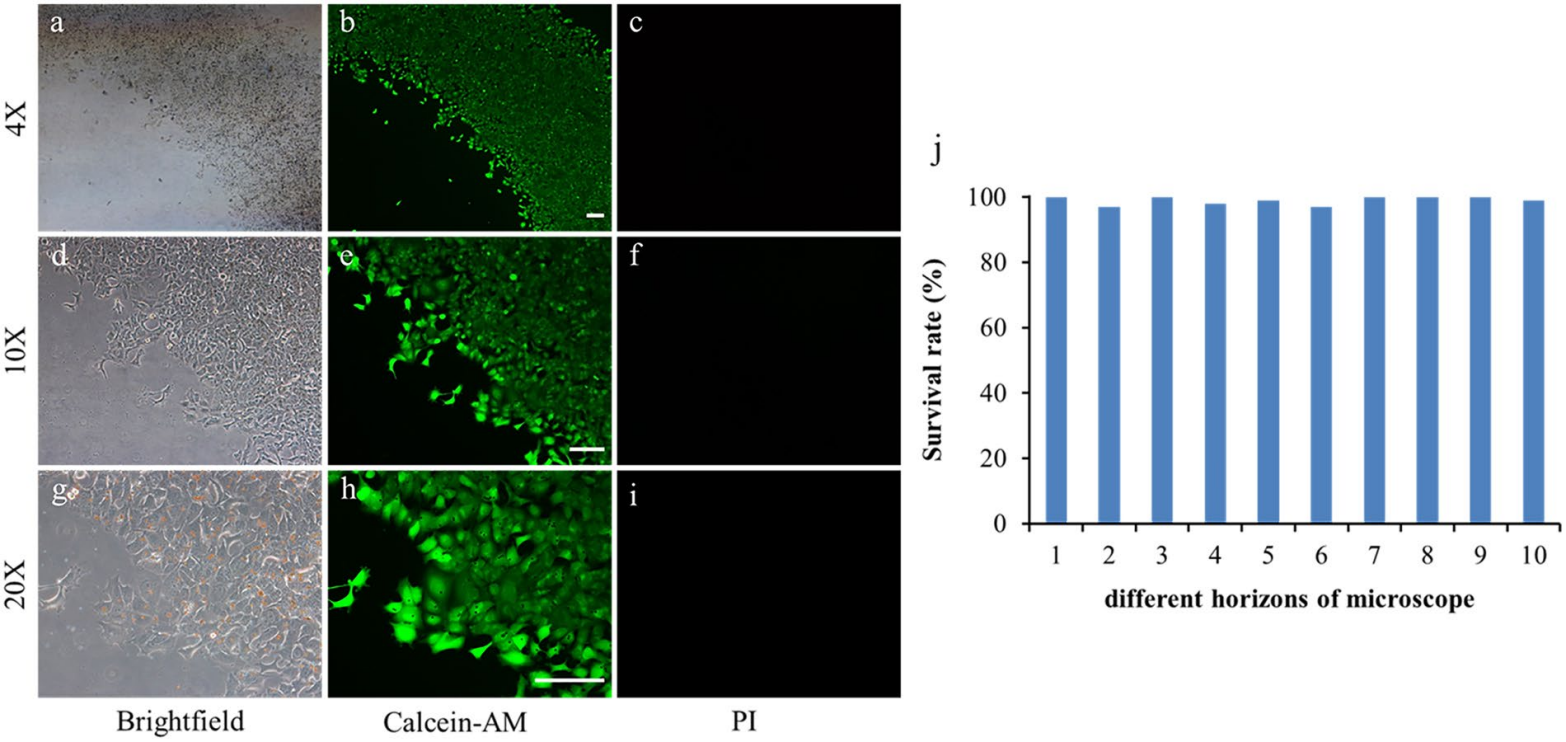
Cell viability assay in the microfluidic channel. (**a**,**d**, and **g**) Bright-field. (**b**,**c**, and **h**) Calcein-AM (green). (**c**,**f**, and **i**) PI (red). (**j**) Survival rate of the cells cultured on the chip. The scale bar is 100 μm.

To successfully culture the cells in the microfluidic channel, surface treatment of the micro-channel and cell seeding are the key points. Therefore, the channels were coated with fibronectin (50 μg/mL) to promote cell adhesion before seeding the cells into the micro-channel. In this chip, the cells were cultured after binding to anti-EpCAM antibody-modified magnetic beads. Even though previous research has demonstrated that these nanoparticles are nontoxic to the cells^33^, cell viability after binding with the magnetic beads was also investigated by staining with Calcein-AM and PI. Calcein-AM produces green fluorescent that can enter the membrane of living cells while PI could not, thus allowing us to determine cell viability. The results were shown in Fig. 3 with 4 × objective magnification for (a-c), 10 × objective magnification for (d-f), and 20 × objective magnification for (g-i). The time point of these images was selected at 8 h after the cells were seeded in the channel. The cells were incubated with dye for 20 min and fluorescent images were captured under a bright field. The results showed that the MCF-7 cells stretched and adhered well in the bright-field pictures and that a vast majority of the adhering cells were stained green and nearly none of the cells were stained red. These results demonstrated that the magnetic beads and PDMS channel did not affect the cell viability during the culture periods, which was similar to previously reported results^33^.

### Feasibility of the microdevice for cell isolation and migration

**Figure 4:**
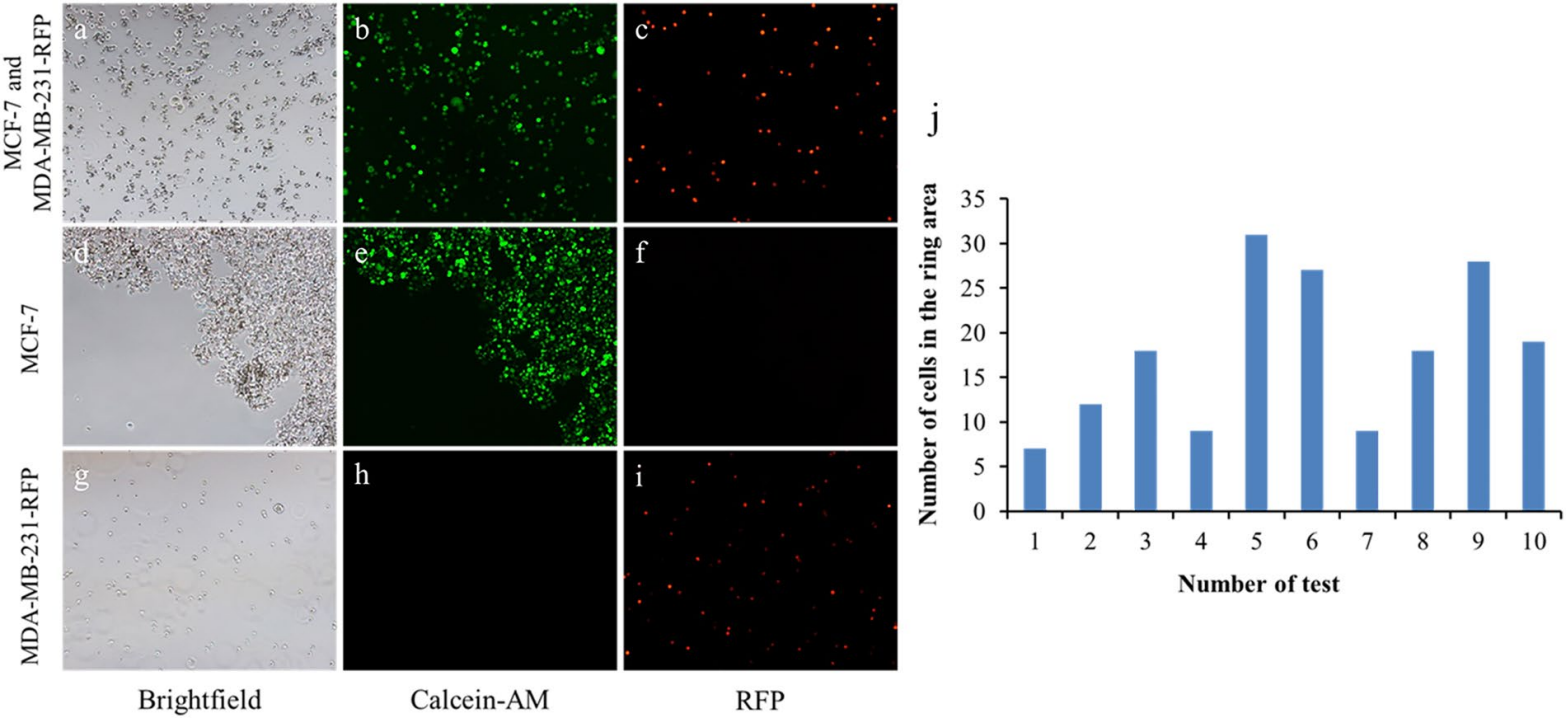
Cell isolation on the device. (**a**–**c**) MCF-7 and MDA-MB-231-RFP cell mixture. (**d**–**f**) MCF-7 cells in the channel after magnet isolation. (**g**–**i**) MDA-MB-231-RFP cells collected at the channel outlet. (**a**,**d**, and **g**) Bright-field. (**b**,**e**, and **h**) Calcein-AM. (**c**,**f**, and **i**) RFP. (**j**) The number of MDA-MB-231-RFP cells remaining in the ring area.

Cell isolation performance is shown in Fig. 4. MCF-7 cells were labelled with the green dye Calcein-AM and mixed with MDA-MB-231-RFP cells. Then, the cell mixture was photographed under microscopy. The bright-field image is shown in Fig. 4a. MCF-7 cells were bound to the magnetic beads, while only a few of MDA-MB-231-RFP cells was bound. After the cell mixture was introduced into the channel and a wash step was performed, the MCF-7 cells were isolated from the MDA-MB-231-RFP cells. The results from Fig. 4d–f and g–I demonstrate that MCF-7 cells formed a ring-shaped pattern, while the MDA-MB-231-RFP cells were collected from the channel outlet. The MCF-7 cells were stained by Calcein-AM dye to reveal their viability, and no obvious differences were observed in their shape and size between the bright-field and green images. These results show that this approach can promote good viability without damaging the cells and successfully perform the cell isolation experiment. In the future, this method and device could be used to isolate cell subtypes from tumour tissue, and investigate the migration behaviour of specific cell subtypes.

**Figure 5:**
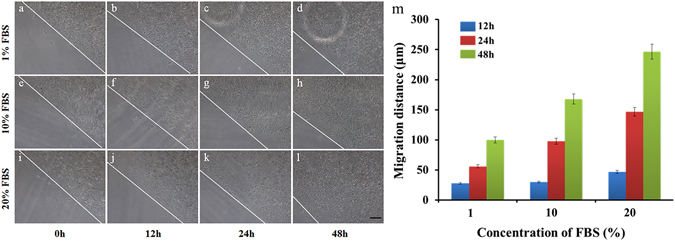
Representative images of MCF-7 cell migration under different FBS concentrations. (**a**–**d**) 1% FBS. (**e**–**h**) 10% FBS. (**i**–**l**) 20% FBS. The images were taken at 0, 12, 24, and 48 h. The lines indicate the initial leading edges at time 0 h and the migration edge after 12, 24, and 48 h. Error bars represent the SD from three different experiments on three devices (n = 3). P < 0.05. The scale bar in the picture represents 100 μm.

MCF-7 cell migration performance after isolation from the MDA-MB-231-RFP cells is shown in Fig. 5. After the isolation step, the MCF-7 cells formed a ring-shaped pattern and were cultured in the channel. The MCF-7 cell migration behaviours under different concentrations of FBS (1%, 10%, and 20%) were tested. As shown in Fig. 5, images of cell migration were taken at different concentrations of FBS at 0, 12, 24 and 48 h. Measurements were made from the initial leading edges. Figure 5 shows that the cell migration speed increases with increased FBS concentrations. The cells located at the circle edge proliferated and migrated towards the circle centre to different degrees under the different FBS concentrations. All the above results indicated that this device could easily perform both cell isolation and the migration assay.

### Effects of TGF-β on MCF-7 cell migration

**Figure 6:**
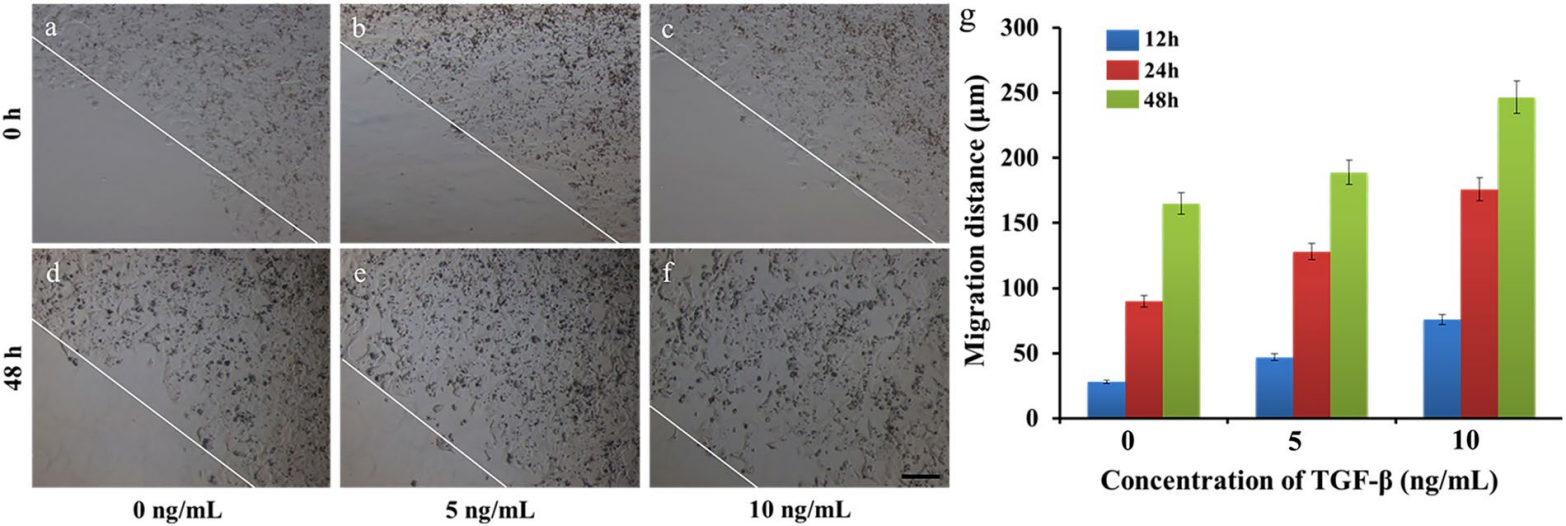
Representative images of MCF-7 cell migration under treatment with different concentrations of TGF-β. (**a** and **d**) Control. (**b** and **e**) 5 ng/mL TGF-β. (**c** and **f**) 10 ng/mL TGF-β. (**g**) Statistical graph for each concentration of TGF-β at different times. The error bars represent the SD from three different experiments on three differebt devices (n = 3). P < 0.05. The images were taken at 0, 12, 24, and 48 h. The lines indicate the initial leading edges at 0 h and the migrated edge after 48 h of migration. The scale bar in the picture represents 100 μm.

Cell migration plays a critical role in various biological processes, including embryogenesis, wound-healing, immune responses, tissue development, and cancer metastasis^34^. To validate the utility of this method as an *in vitro* model for migration, we investigated MCF-7 cell migration towards tumour growth factor (TGF-β). TGF-β is a major inducer of EMT. Studies have demonstrated that MCF-7 cells were induced into EMT by TGF-β, which also promotes MCF-7 cell migration^35^. As shown in Fig. 6, MCF-7 cells were treated with TGF-β at different concentrations (0, 5 and 10 ng/mL) for 48 h, cells migrated 160 μm when no TGF-β was present in the medium, though the cell migration distance increased to 240 μm upon addition of 10 ng/mL TGF-β into the medium (Fig. 6g). These results showed that migration distance of MCF-7 cells was increased with increased concentrations of TGF-β.

**Figure 7:**
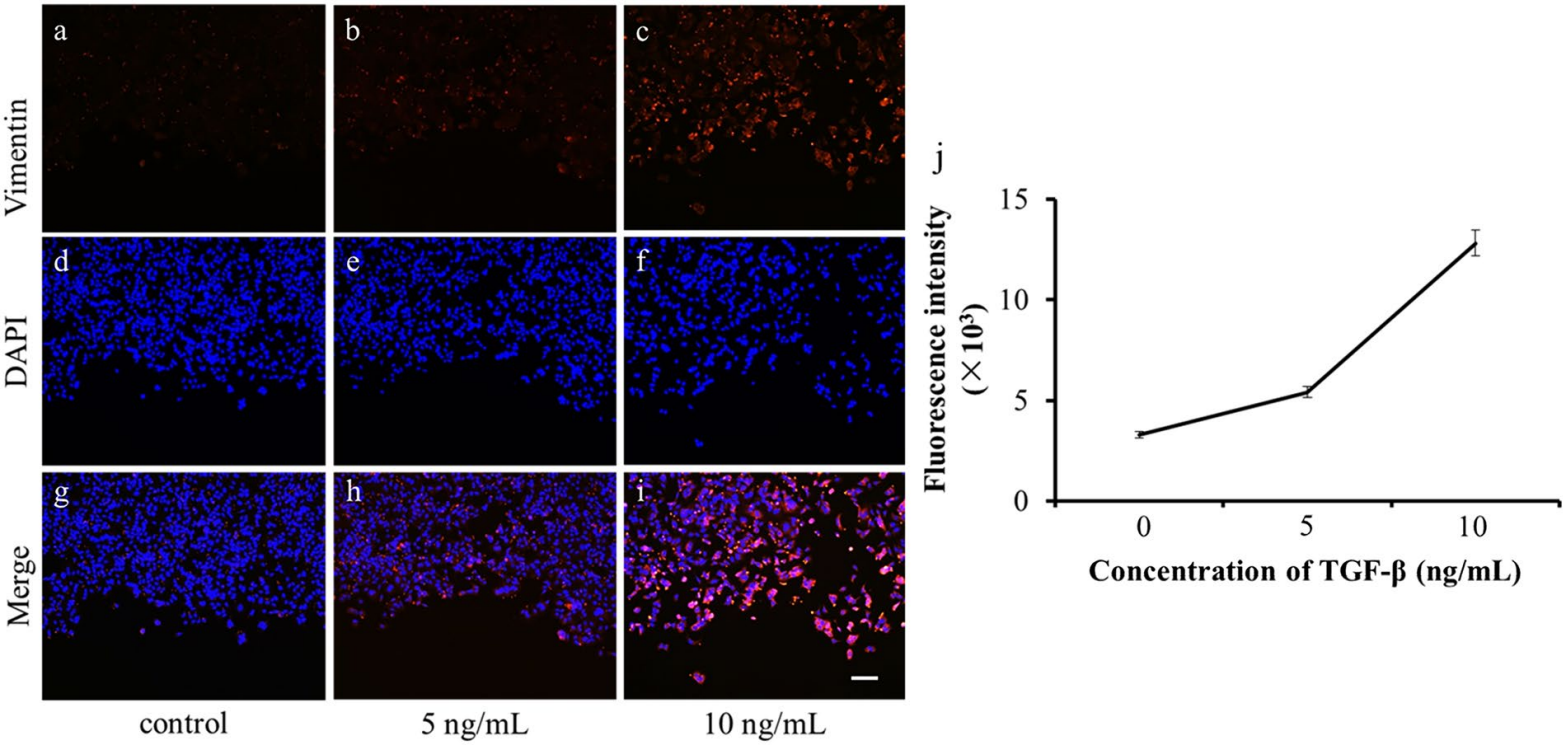
The effect of TGF-β on vimentin expression. (**a**–**c**) Cells were fixed and stained with anti-vimentin antibody (red) after treatment with different concentrations of TGF-β (0, 5 and 10 ng/mL). (**d**–**f**) Cells were fixed and stained for nuclei with DAPI (blue) after treatment with different concentrations of TGF-β (0, 5 and 10 ng/mL). (**g**–**i**) Merged images for a-f. (**a**,**d**, and **g**) Control. (**b**,**e**, and **h**) 5 ng/mL TGF-β. (**c**,**f**, and **i**) 10 ng/mL TGF-β. (**j**) Fluorescent intensity of vimentin. The scale bar in the picture represents 100 μm.

The chip could also be used to perform on-chip immunostaining directly after a migration assay. During the EMT process, expression of the epithelial markers is decreased, whereas the expression of mesenchymal markers increases^36^. TGF-β influences the EMT by activating the expression of mesenchymal marker such as vimentin^37^. Therefore, in this experiment, cells were stained with an anti-vimentin antibody and DAPI after MCF-7 cell migration assay with TGF-β at different concentrations (0, 5, and 10 ng/mL) for 48 h. The fluorescence intensity of vimentin (Fig. 7, in red) represents the vimentin levels. Vimentin and nuclear fluorescence images of the cells under each experimental condition obtained at 10 × magnification were overlaid. The results show that vimentin expression increased with increasing TGF-β concentration. In other words, TGF-β treatment of MCF-7 cells led to EMT, as judged by the up-regulation of the mesenchymal marker vimentin. These results were similar to previously reported results using a conventional method. However, the changes in protein expression were a complex and slow process during EMT. Thus, further investigations should be performed to indicate the cell protein changes during EMT.

## Conclusion

The integrated microfluidic chip is easy to fabricate with common materials and includes a magnet adapter for supporting the six magnet ring, a PDMS channel for medium control, and a glass slide for culturing cells. The cells formed a ring-shaped pattern under the induced force of the magnetic field after being isolated from the cell mixture. Then, the migration assay was easily performed on the chip. Compared with the wound-healing assay, our method could isolate specific cells and investigate their migration abilities. Additionally, device consumption was greatly reduced. The above results demonstrated that the integrated microfluidic cell isolation and migration chip will help researchers to better understand MCF-7 migration under TGF-β stimulation. Moreover, the relationship between other cells and biological factors in a biomimetic microenvironment could also be investigated, which could allow researchers to better understanding cell subtype migration mechanisms in complex tumour tissues.

Many cell isolation and migration microfluidic chips and other devices have been reported in recent years^6–11, 22–31^. Many microfluidic chip structures have been designed and fabricated to display the advantage of microfluidics no regardless of whether they include large numbers of cells or a single cell. However, to date, there is not a device that investigates the migration of a group of cells that express a specific marker form a cell mixture^10^. Thus, our microfluidic chip could capture epithelial MCF-7 cells from a cell mixture that included MDA-MB-231-RFP cells with antibody-modified magnetic beads and investigate EMT biological processes under stimuli. The effect of TGF-β on MCF-7 cells was explored and indicated that TGF-β could promote MCF-7 migration, leading to EMT as judged by the up-regulation of the mesenchymal marker vimentin. Further work aims to isolate mesenchymal cells from tumour tissue samples and investigate their migration abilities under different stimuli. We have also tried to use a cellphone to capture images at different time because enough of the magnetic beads are megascopic^32^. This is a novel method that can integrate cell isolation and migration on a chip. We believe that our approach is a reliable method due to its simplicity and low cost for subtype cell migration studies on tumour tissue samples.

## Materials and Methods

### Materials

Human fibronectin was purchased from Millipore (USA). The cell culture medium, foetal bovine serum (FBS), trypsin/EDTA solution and phosphate-buffered saline (PBS) were purchased from Gibco Invitrogen Corporation (USA). Hydralazine hydrochloride (CAS: 304-20- 1) 4% paraformaldehyde solution, Triton X-100, and bovine serum albumin (BSA) were purchased from Sigma-Aldrich (USA). TGF-β was purchased from PeproTech. Alexa Fluor 546 goat anti-rabbit secondary antibody and 4′,6-diamidino-2-phenylindole (DAPI) were purchased from Invitrogen (USA). Anti-vimentin antibody was purchased from Abcam (USA) and photoresist (Ruihong 304) with developer was purchased from Suzhou Ruihong Electronic chemicals Co., LED, China. Poly- (dimethylsiloxane) (PDMS) was purchased from Dow Corning (USA); the kit is called the Sylgard 184 kit.

### Chip fabrication and operation

A circular channel with a radius and height of 4 mm and 80 μm, respectively, was used in this work. The desired channel pattern was drawn using the L-edit software (Tanner Research, Inc.) and printed on glass to be a mask version. The silicon mould for the magnetic-based cell migration assay chip was manufactured using standard lithography techniques with a RZJ-304 resist; the detailed fabrication protocol for the PDMS channel are the follows. A polished silicon wafer was washed with aqua regia and spin-coated with a 3-μm thick photoresist layer, followed by exposure and a developing process. The channel pattern was left on the wafer and the 80-μm deep mould was etched by deep reactive ion etching (DRIE) using the photoresist as an etch mask to develop the silicon mould (Fig. S1a). Finally, the mould was immersed in acetone, sonicated for 5 min, rinsed with ethanol and deionised water and dried by nitrogen gas. Before pouring the PDMS on the mould, the mould surface was pre-treated with trichloro (1 H, 1 H, 2 H, 2H-perflurooctyl) silage in a desiccator under vacuum. The silicone elastomer PDMS (Sylgard 184) contains a polymer base and curing agent that were then mixed at a ratio of 10:1 (weight), poured onto the mould and cured in a drying oven at 80 °C for 1 h. Then, the PDMS chip was peeled from the mould and cut into 75 mm × 25 mm pieces (Fig. S1b). Inlet and outlet holes were punched and the chip was bonded to a standard glass slide following treatment with oxygen plasma (PDC-32G, Harrick Scientific Products, Inc.), which resulted in the complete microfluidic chip (Fig. 2b). The chip’s magnetic adapter with a circular hole with a 4-mm radius and 1.3-mm height was fabricated from polytetrafluoroethylene (Fig. S1c). The ring magnet was placed into the circular hole and formed into the magnet adapter (Fig. S1d). The chip was placed onto the magnetic adapter after loading the cells into the channel, and both of them were placed into a cell culture incubator until the cells adhere to the inner-surface of the chip (Fig. 2e). The entire microfluidic chip is shown in Fig. 2f.

### Cell culture and sample preparation

The human breast cancer cell lines MCF-7 and MDA-MB-231-RFP were obtained from the China Infrastructure of Cell Line Resource. Cells were maintained in high-glucose Dulbecco’s Modified Eagle Medium (DMEM; Gibco) supplemented with 10% foetal bovine serum (FBS; Gibco), penicillin (100 U mL-1, Gibco) and streptomycin (100 μg mL-1, Gibco). The cells were cultured at 37 °C in a humidified incubator with 5% CO_2_. After reaching ~80% confluence in the culture dish, the cells were removed with 0.25% trypsin/EDTA and resuspended in 1 mL 1% BSA/PBS solution. The MCF-7 and MDA-MB-231-RFP cells were mixed at a ratio of 5:1, incubated with Dynabeads® Epithelial Enrich (Invitrogen) at 4 °C for 20 min, washed three times, resuspended with freshly supplemented 10% FBS DMEM medium to a density of 1 × 10^6^ cells/mL and then seeded into the chip. Before cell seeding, the chips, tubes, adapter and magnets were autoclaved for 20 min at 121 °C. Additionally, the channels were coated with fibronectin (50 μg/mL) to promote cell adhesion. The chips were then incubated for at least 1 h in a 37 °C incubator before the chip was washed with PBS. Finally, the chip was dried in a clean hood at room temperature.

### Cell viability assay

Cell viability assessment in the microfluidic channel was performed using Calcein-AM and PI staining^32^. After the migration assay on the chip, the medium was removed, and the channel was washed twice with PBS. Then, 10 μg/mL of Calcein-AM and PI were added into the channel for 20 min f staining at 37 °C. Next, PBS was introduced to wash the channel. Finally, the chip was observed and photographed under the microscopy. The number of live and dead cells was calculated from the stained cells in the Calcein-AM and RFP images.

### On chip cell staining

The MCF-7 cells in the chip were stained for vimentin and the nuclei using anti-vimentin antibody (primary antibody) and Alexa Fluor 546 goat anti-rabbit secondary antibody and DAPI, respectively. The anti-vimentin antibody was diluted 1:500 in 1% BSA/PBS solution and the Alexa Fluor 546 goat anti-rabbit secondary antibody was prepared at a concentration of 10 μg/mL in 1% BSA/PBS solution. The working concentration of DAPI was 0.2 μg/mL in a BSA/PBS solution. All of the solutions were infused into the channels using 1 mL syringes (Fig. S2). The cells were gently washed with 1 mL PBS, fixed with 4% paraformaldehyde for 10 min at room temperature, permeabilized with 0.1% Triton X-100 for 5 min, and blocked with 1% BSA for 1 h. Then, the fixed cells were stained with 500 μL of anti-vimentin antibody and incubated at 37 °C for 1 h. Next, 500 μL of Alexa Fluor 546 goat anti-rabbit secondary antibody solution was added to the fixed cells and incubated at room temperature for 1 h, after which 500 μL of DAPI solution was infused into the channel for 2 min at room temperature. The channel was washed with 1 mL PBS after each step.

### Microscopy and image analysis

An inverted microscope (Ti-s, Nikon, Japan) with a CCD camera (Ds-Ri1, Nikon, Japan) was used to acquire the phase contrast and fluorescent images. The capture position of the images is shown in Figure S3 (yellow solid line). Data acquisition of the cell migration distance was determined from the average values from three different devices. The fluorescent intensity of the staining image was processed with ImageJ. Significance was defined as p < 0.05. All the statistical analyses were performed with OriginPro. Error bars in the figures represent the standard deviation.

## Electronic supplementary material

An integrated method for cell isolation and migration on a chip
