# Supplementary material for: An integrated method for cell isolation and migration on a chip
Source: Sci Rep. 2017 Aug 21;7:8963. doi: 10.1038/s41598-017-08661-z (PMC5566426; doi:10.1038/s41598-017-08661-z)
Supplement: Supplementary file 1 — An integrated method for cell isolation and migration on a chip [file 41598_2017_8661_MOESM1_ESM.pdf]

Supplement material information

An integrated method for cell isolation and migration on a chip

Xiaoqing Lv[1,4], Zhaoxin Geng[2]*, Zhiyuan Fan[1,4], Shicai Wang[3], WeiHua Pei[1] and Hongda Chen[1]

1 State Key Laboratory of Integrated Optoelectronics, Institute of Semiconductors, Chinese Academy of Sciences, Beijing, China

[2] School of Information Engineering, Minzu University of China, Beijing, China

[3] State Key Laboratory of crystal materials, Shandong University, Jinan, China

[4] University of Chinese Academy of Sciences, Beijing, China

*corresponding author: Zxgeng@muc.edu.cn

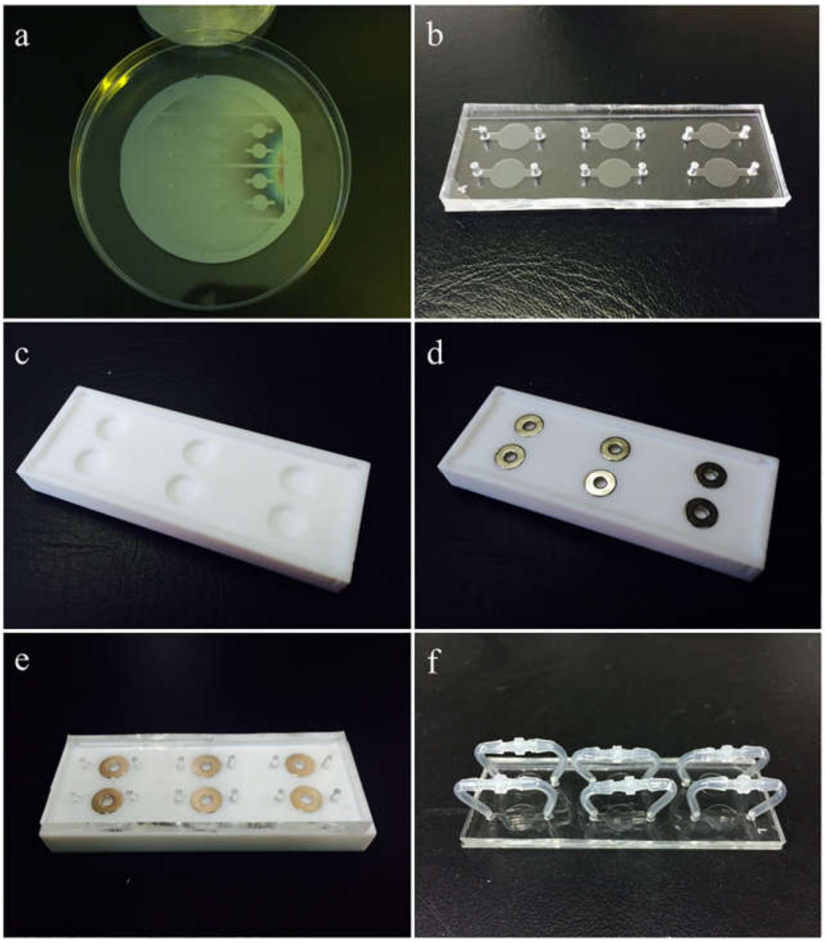

Figure S1. Set up of the magnetic-based cell migration chip. (a) Silicon wafer. The wafer was treated with trichloro (1H,1H,2H,2H-perflurooctyl) silane to prevent PDMS adhering to the wafer. (b) PDMS chip. The radius of the circle channel was 4mm and the height was 80μm. (c) Ring magnet adapter. Radius of the channel was 4mm, height was 1mm. (d) Magnet ring. Radius of the outer ring was, radius of the inner ring was 1.5mm, height of the magnetic ring was 1mm. (e) Whole structure of the magnetic-based cell migration chip; (f) Chip with tube of inlet and outlet which consist the whole microfluidic chip.

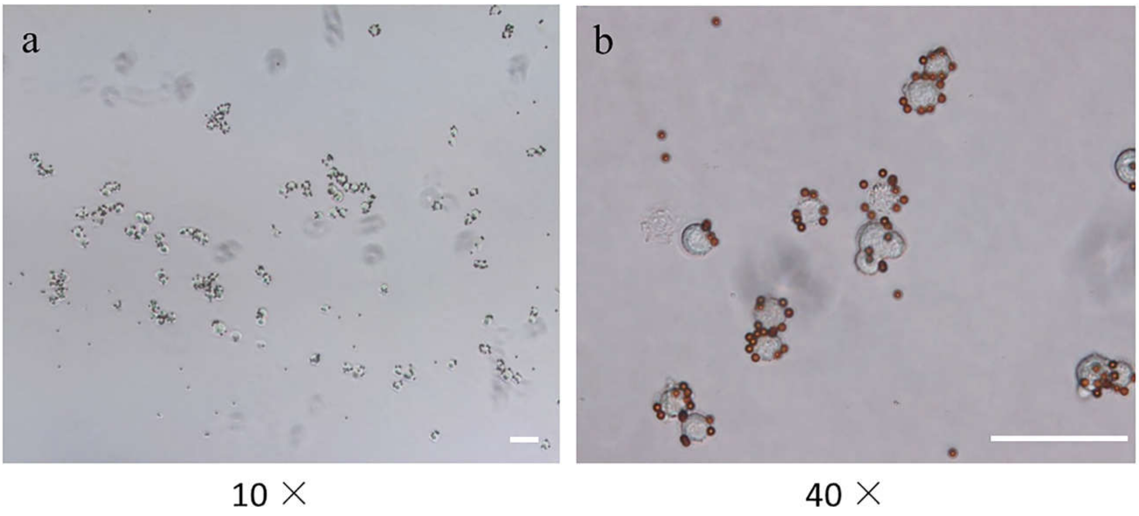

Figure S2. Image of cells cultured with magnetic beads. (a) The objective magnification was 10×. (b) The objective magnification was 20×. The scale bar is 100μm.

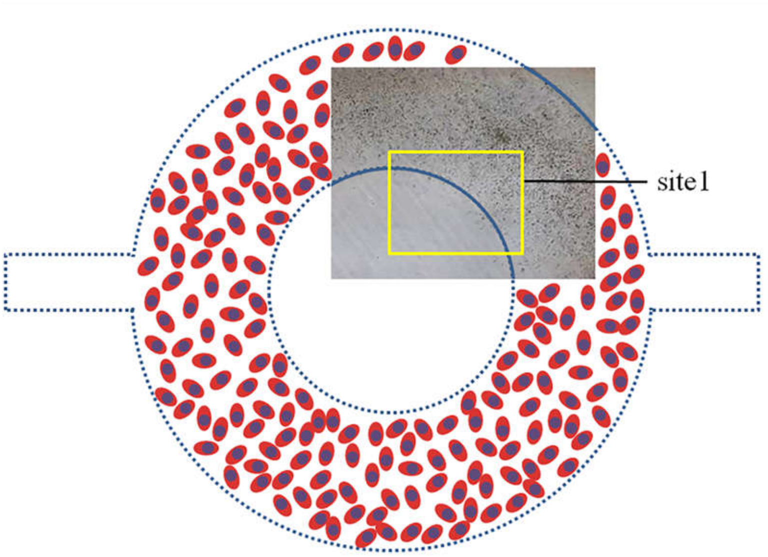

Figure S3. Schematic of image capture. Blue dotted lines represent the circle edge; yellow solid line represents the image capture location.

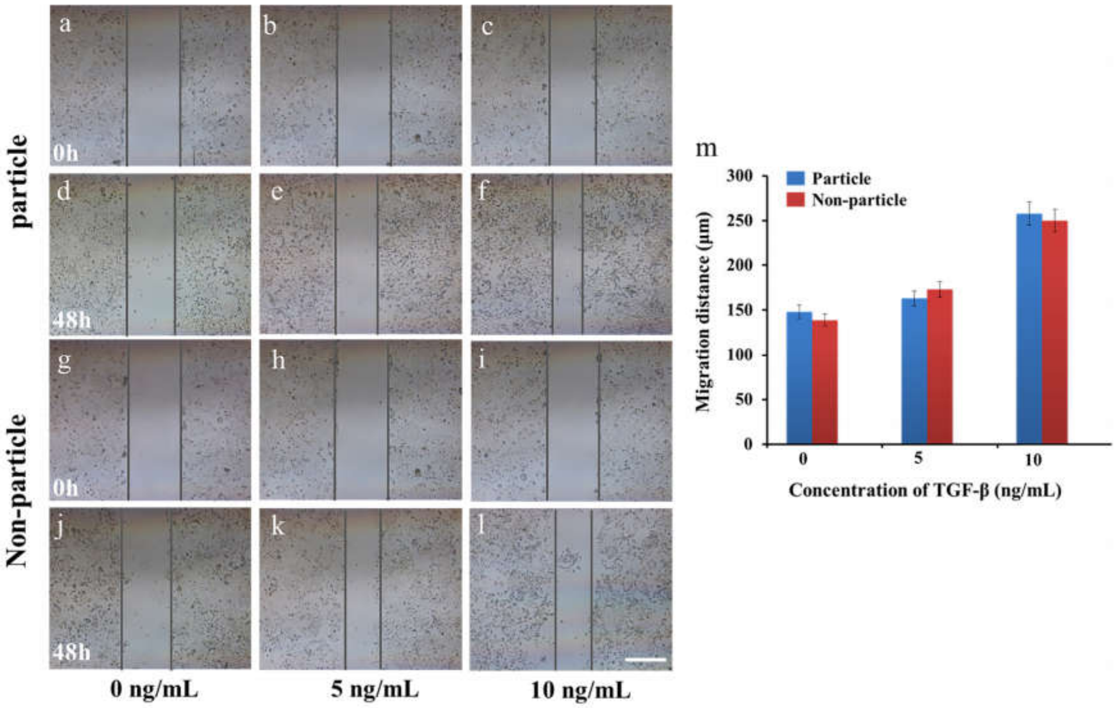

Figure S4. Representative images of MCF-7 cell migration distance with and without magnetic beads. (a-f) MCF-7 cell with magnetic beads. (g-l) MCF-7 cell without magnetic beads. (m) The quantitative graph of migration distance under different concentration of TGF-β. Error bar epresented the SD from three ifferent experiments in three devices (n=3). $P < 0.05$.The scale bar in the picture represents 500μm.

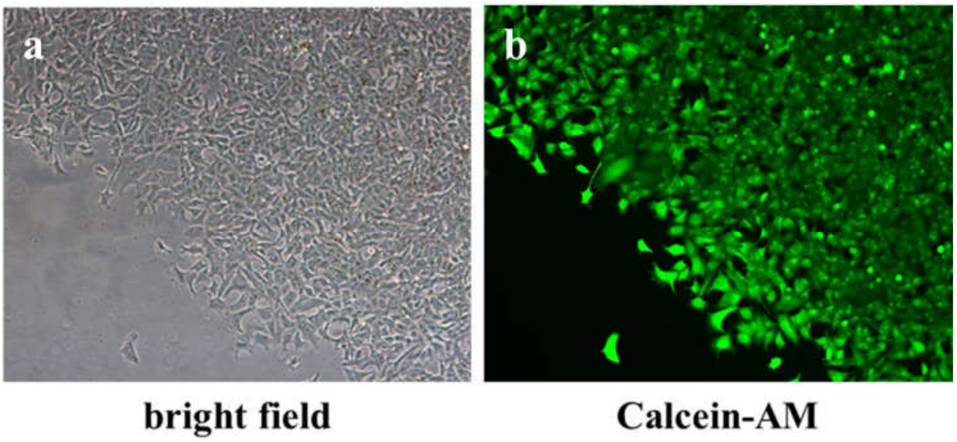

Figure S5. Calcein-AM staining image of MCF-7. (a) Bright field. (b) Calcein-AM (green).
